# Donor-site closure using absorbable dermal staple for deep inferior epigastric artery perforator flaps: its efficacy and cosmetic outcomes

**DOI:** 10.1186/s40064-016-1988-9

**Published:** 2016-03-23

**Authors:** Hyun Ho Han, Seong Yeon Kim, Yoon Jae Lee, Suk Ho Moon, Deuk Young Oh

**Affiliations:** Department of Plastic Surgery, Seoul St. Mary’s Hospital, College of Medicine, The Catholic University of Korea, 222 Banpo-daero, Seocho-gu, Seoul, 16591 Republic of Korea

**Keywords:** Absorbable dermal staples, Deep Inferior epigastric artery perforator flap, Donor site, Operation time, Scar

## Abstract

**Background:**

Surgeons tend to pay less attention to the donor site during breast reconstruction using deep inferior epigastric artery perforator flaps because attention is focused on microanastomosis and breast shaping. Therefore, donor site closure is typically performed by a secondary operator. We present consistently reduced operative times and improved scar quality using an absorbable dermal staple.

**Methods:**

Retrospective review was performed on 25 patients who were either standard suture controls (group I, n = 15) or received absorbable staples (group II, n = 10). Mean age, flap size, whole operative time, and length of hospital stay were collected. The donor site scar was evaluated by three plastic surgeons in a blinded manner using the modified Vancouver scar scale 6 months after surgery. Data were analyzed with the independent *t* test, and a *p* value ≤0.05 was considered significant.

**Results:**

No differences were detected between the groups for age, harvested flap size, or length of hospitalization. However, operative time was significantly longer in group I (1.07 ± 0.24 min/cm^2^) than that in group II (0.86 ± 0.16 min/cm^2^, *p* = 0.015). The total scar assessment score was significantly lower in group II (3.8 3 ± 1.30) than that in group I (5.27 ± 1.83, *p* = 0.043).

**Conclusions:**

Absorbable dermal stapling reduced operative time, compared to that of traditional suturing. In addition, scar quality from absorbable dermal staples was superior to that resulting from traditional sutures.

**Level of evidence:**

II.

## Background

Surgeons, particularly with plastic surgeons, prefer efficient closure for long surgical incisional wounds and expect high quality scar outcomes. However, wound closure can proceed too quickly without attention to approximating the wound margin, resulting in unpreventable scarring. In contrast, if closure is performed too carefully, the surgery can become too long.

Surgeons tend to pay less attention to the donor site during breast reconstruction using deep inferior epigastric artery perforator (DIEP) flaps as they are concentrating on the micro-anastomosis and breast shaping. Furthermore, a secondary operator is often responsible for the lengthy closure procedure of a long incisional wound for a multi-layered donor site. This time penalty may be associated with the suture skill of the secondary operator, leading to an inconsistent donor site or final scar shape. Additionally, there is an increased risk for a needle stick injury, inflammation, or infection due to longer needle handling time and increased tissue irritation (Mehta et al. 1996; Setzen and Williams 1997).

INSORB dermal staples (Incisive Surgical, Plymouth, MN, USA) are composed of 70 % polylactic acid and 30 % polyglycolic acid and are absorbed into the body through hydrolytic processes 3–4 weeks postoperatively (Duteille et al. 2013; Herridge 2009). Additionally, dermal staples hold the skin inside the dermal layer of the incisional line and are not outwardly visible. Few studies have reported outcomes of using absorbable INSORB staples. One such study revealed less histological inflammation from absorbable INSORB staples compared to that of suturing in a pig model (Fick et al. 2005). A human-based study reported 4–5 times faster closure of abdominal and breast wounds using absorbable INSORB staples than that of suturing (Duteille et al. 2013; Cross et al. 2009).

In this study, we determined the outcomes of using INSORB dermal staples to close the donor site after elevating DIEP flaps. We hypothesized that INSORB staples would shorten operative time and result in better scar outcomes at the long-term follow-up than those of traditional suture closure.

## Patients and methods

Our institutional review board approved this study. A total of 25 patients were enrolled from January 2014 to December 2014 based on a retrospective chart review and divided into a traditional suture group (n = 15) and an absorbable INSORB staple group (n = 10). The 15 patients in the suture group were enrolled separately from those in INSORB group. Mean age, flap size, operative time, and length of hospital stay were collected. Most procedures were performed by a single surgeon (Oh DY), including elevation of the DIEP flap, donor site closure, microvessel anastomosis, and flap inset and shaping.

### Operative technique

Abdominal closure following harvest of a DIEP flap for breast reconstruction was performed under hip flexion to reduce tension. The deep fat layer was sutured with 1–0 Vicryl sutures at 5–6-cm intervals in both groups. Subsequently, group I received 3–0 Vicryl dermal sutures at 2–3-cm intervals, and INSORB dermal staples were used in group II. Subcuticular 4–0 polydioxanone sutures were used in both groups, and the skin suturing was not performed independently.

### INSORB technique

**Fig. 1:**
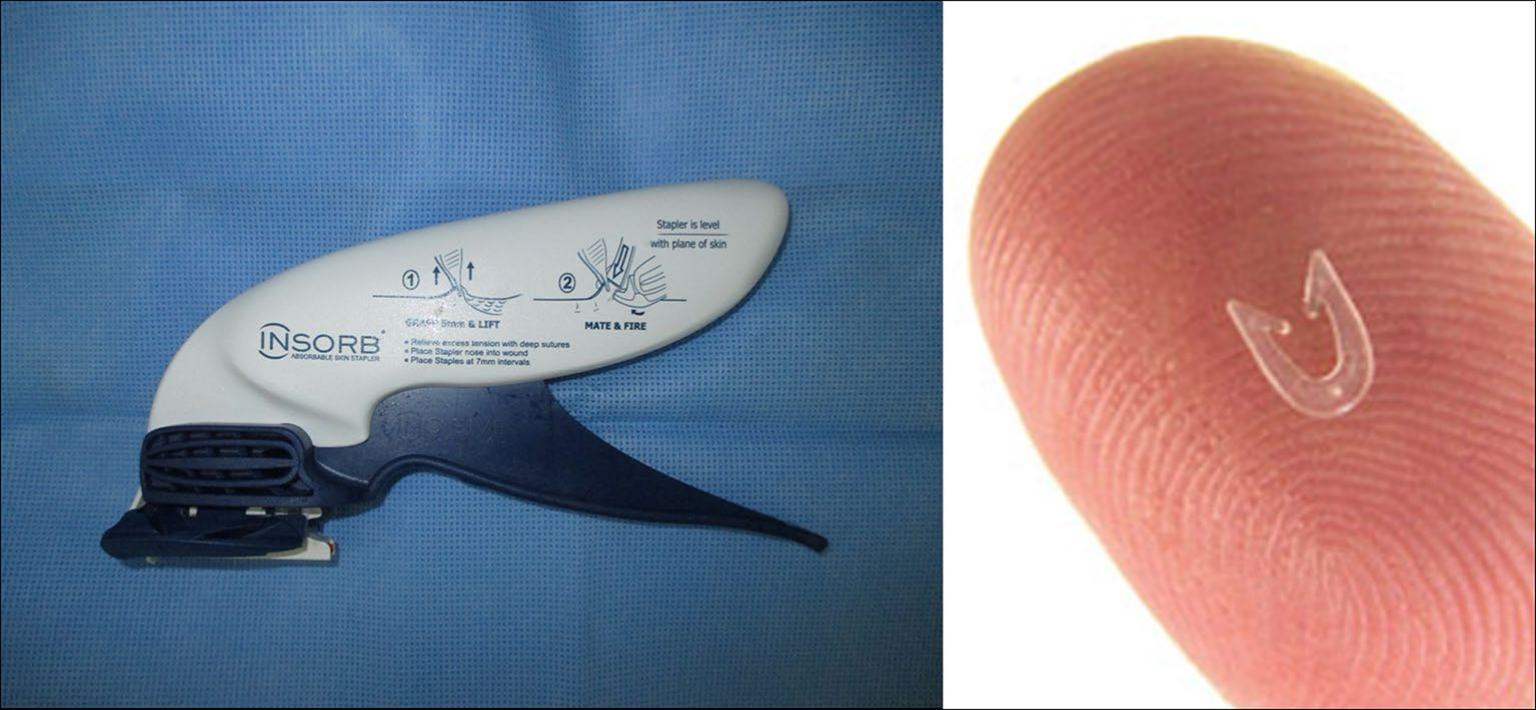
*Left* Absorbable dermal stapler. *Right* C-shape INSORB dermal staples (Incisive Surgical, Plymouth, MN, USA), composed of 70 % polylactic acid and 30 % polyglycolic acid

**Fig. 2:**
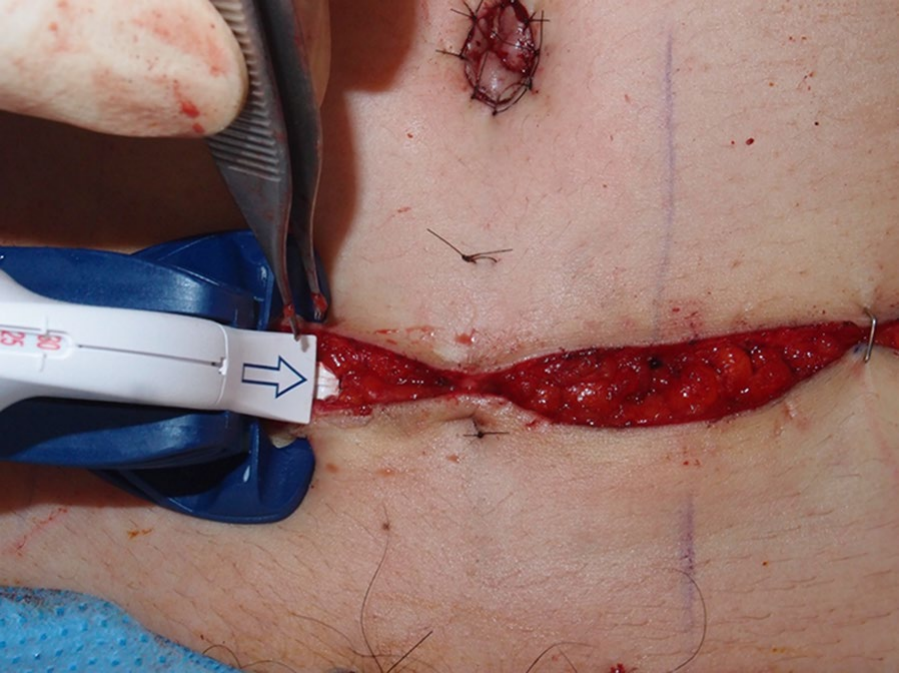
Using the stapler tip to capture the dermal layer on both sides of the incision

INSORB dermal staples have two heads (Fig. 1, left) and a U-shaped form (Fig. 1, right). The staples were applied to the dermal tissue layer. Both sides of the skin margin were pushed into the head of staples using teeth forceps, approximated, and the closure performed while the wound was maintained in an everted state (Fig. 2).

### Postoperative care

Patients in both groups were kept supine in hip flexion for 2 days postoperatively to reduce tension on the abdomen following closure of the DIEP flap donor site. They were ambulated in a waist-flexion state for 7 days and remained upright thereafter. Steri-strip dressing (3M, Inc., St. Paul, MN, USA) was used as skin tape for 3 weeks postoperatively, and a maternity belt was prescribed for 1 month following the operation.

### Scar assessment

The DIEP donor site scar was evaluated 6 months postoperatively when the nipple or nipple–areolar complex was formed, as this was considered the completion of scar maturation. The donor site scar was evaluated on clinical photographs by three blinded plastic surgeons using the modified Vancouver scar scale (Table 1) (Truong et al. 2007; Fraser et al. 2005). Details of vascularity (0–3), pigmentation (0–2), and height (0–3) were evaluated, and mean scores were calculated. Pliability details in the Vancouver scar scale were not considered, as they were not interpretable by photography.

**Table 1:** Modified Vancouver scar scale

Scar characteristic	Score
Vascularity	
Normal	0
Pink	1
Red	2
Purple	3
Pigmentation	
Normal	0
Hypopigmentation	1
Hyperpigmentation	2
Height	
Flat	0
<2 mm	1
2–5 mm	2
>5 mm	3

### Statistical analysis

Data were compared using the independent *t* test. A *p* value ≤0.05 was considered significant.

## Results

Results for mean age, flap size, operative time, and length of hospitalization are shown in Table 2. Mean patient ages were 45.20 ± 8.94 years in group I and 49.9 ± 6.21 years in group II. Flap sizes were 562.08 ± 117.54 cm^2^ in group I and 495 ± 79.2 cm^2^ in group II. Hospitalization durations were 9.33 ± 0.82 days for group I and 9.90 ± 0.31 days for group II (all *p* > 0.05). Operative time was reduced substantially in group II (418.00 ± 51.49 min) compared to that in group I (490.87 ± 87.63, *p* = 0.027). Additionally, a significant difference in operative time per flap size was observed (1.07 ± 0.24 min/cm^2^ in group I and 0.86 ± 0.16.49 min/cm^2^ in group II, *p* = 0.015).

**Table 2:** Intraoperative results

	Group I (suture)	Group II (absorbable stapler)	*p*
Mean age	45.20 ± 8.94	49.9 ± 6.21	0.163
Flap size (cm^2^)	562.08 ± 117.54	495.80 ± 79.28	0.133
Operation time (min)	490.87 ± 87.63	418.00 ± 51.49	0.027
Hospital day	9.33 ± 0.82	9.90 ± 0.31	0.133
time/flap size (min/cm^2^)	1.07 ± 0.24	0.86 ± 0.16.49	0.015

**Fig. 3:**
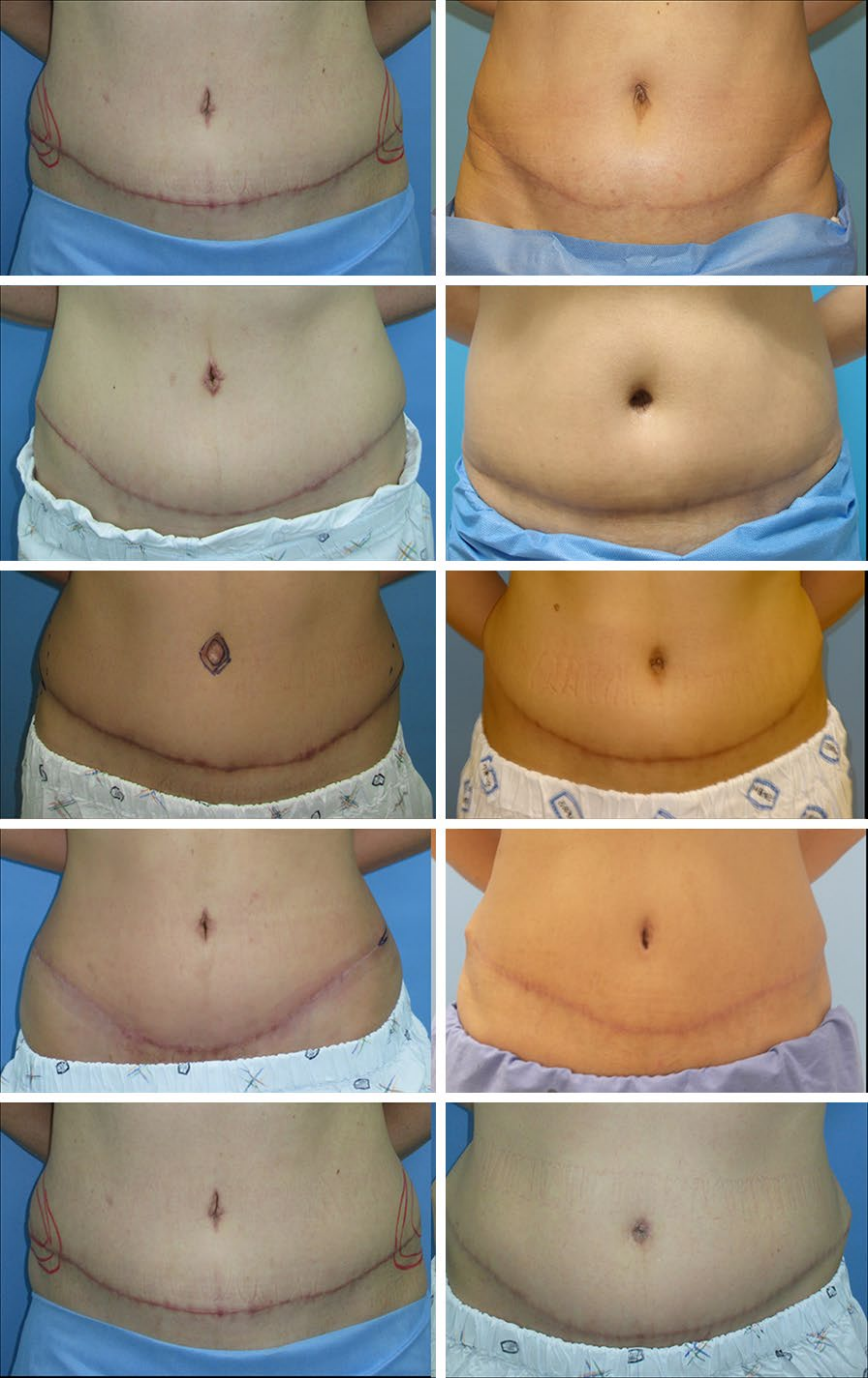
Six month postoperative photographs. *Left row* group I, *right row* group II

The scar assessment results are presented in Table 3. The total score was significantly less in group II (2.50 ± 0.92) compared to that in group I (3.51 ± 1.30, *p* = 0.044) (Fig. 3). Group II showed tended to have better vascularity and height scores. In contrast, significantly different pigmentation scores were observed between groups I (1.76 ± 0.35) and II (1.33 ± 0.54, *p* = 0.026).

**Table 3:** Scar assessment results

	Group I (suture)	Group II (absorbable stapler)	*p*
Vascularity (0–3)	0.86 ± 0.72	0.57 ± 0.52	0.268
Pigmentation (0–2)	1.76 ± 0.35	1.33 ± 0.54	0.026
Height (0–3)	0.88 ± 1.83	0.60 ± 0.35	0.16
Total (0–8)	3.51 ± 1.30	2.50 ± 0.92	0.044

## Discussion

Absorbable INSORB stapling is a straightforward technique that can be mastered with limited experience. Therefore, INSORB staples can be used by an inexperienced secondary operator with consistent results, while the skillful operator concentrates on the micro-anastomosis of the breast site after elevating the DIEP flap. We show shortened operative times using the stapler during a time-consuming operation, which is advantageous for wound management and to ensure good scar results. Using the INSORB stapling technique provides cost-benefits by effectively shortening total operative time.

Wound closure using INSORB has been the subject of a number of studies. In 2013, Duteille et al. (Duteille et al. 2013) reported reduced closure time during breast and abdominal surgeries. In 2009, Cross et al. (2009) evaluated operative time and scarring outcomes during breast wound closure in patients undergoing breast reconstruction with a tissue expander in a prospective, randomized, controlled study. However, scarring was evaluated 4 months postoperatively prior to complete scar maturation, even resulting in no meaningful scar outcome results.

In contrast, we evaluated scars 6 months postoperatively when the nipple or nipple–areolar complex had formed. Minimal loss to follow-up, an appropriate scar evaluation timeline, and good results from a high-tension DIEP donor site are meaningful changes made in the current study.

In addition, we were encouraged to obtain good scar results in an East Asian patient exhibiting Fitzpatrick skin type IV–V, which is often vulnerable to hypertrophic and keloid scarring, compared to that of Caucasians (Fitzpatrick 1988).

Some hypotheses can be proposed following the positive results we obtained from Vancouver scar scale evaluation. First, we closed wound sites using an atraumatic technique with absorbable INSORB staple, which reduced skin irritation and skin margin handling time. Second, the Vicryl used in the control group was braided absorbable thread; thus, there was a higher probability of infection due to inflammation and bacterial colonization of the absorbable thread (Piñeros-Fernandez et al. 2006). This was mitigated by selecting different thread for the control group. Last, the stapling apparatus has strong holding tension and may help with prolonged wound approximation. Biedrzycki et al. (2015) reported that absorbable staples can bear much greater tension of first failure compared with that of metallic staples or thread. Based on these observations, absorbable staples are assumed to approximate the wound margin more strongly than thread.

Not all scars of patients sutured with absorbable staples were excellent and individual differences were observed. Figure 3 shows that patient 5 in group II did not obtain a good result, but overall scar quality of group II was better than that of group I.

An interesting point was the significant difference between the groups on the pigmentation category of the Vancouver scar scale. Hyperpigmentation may be attributable to the inflammation phase of the wound healing process (Chadwick et al. 2012). Although the exact mechanism is unknown, increased melanocyte activity results from elevated levels of prostanoids, cytokines, chemokines, other inflammatory mediators, and reactive oxygen species released during inflammation (Davis and Callende 2010). Therefore, as described previously, INSORB may result in less pigmentation closer to that of normal skin, compared with that of Vicryl sutures, due to mitigated wound inflammation.

This study had a number of limitations; the study design was not prospective, the number of patients in the two groups was not consistent, and the total number of patients was small. The reduced operating time may be the result of other non-suture variables, such as the assistant or operator and the vascular condition or bleeding tendency of the patient. Unfortunately, alternative absorbable suture material, particularly monofilament, was not used to compare with Vicryl sutures, and the pathology was not confirmed directly.

In conclusion, absorbable INSORB staples provided a convenient way to reduce operative time compared to that of traditional sutures. In addition, scar quality from the absorbable INSORB staples was better than that from traditional sutures.
